# Senolytics in the treatment of diabetic retinopathy

**DOI:** 10.3389/fphar.2022.896907

**Published:** 2022-08-26

**Authors:** Jannah Waled Hassan, Ashay D. Bhatwadekar

**Affiliations:** Department of Ophthalmology, Indiana University, Indianapolis, IN, United States

**Keywords:** diabetic retinopathy, senolytics, diabetes, inflammation, pharmacological agents

## Abstract

Diabetic retinopathy (DR) is the most common complication of diabetes. DR is characterized by damage to retinal vasculature resulting in vision impairment and, if untreated, could eventually lead to blindness. The pathogenic mechanism of DR is complex; emerging studies suggest that premature senescence of retinal cells and subsequent secretion of inflammatory cytokines exacerbate DR disease state by stimulating paracrine senescence, pathological angiogenesis, and reparative vascular regeneration. Senolytics are a new class of drugs that can selectively clear out senescent cells from the retina, thus holding a significant promise in DR treatment and prevention. In this review, we discuss the critical role of cellular senescence in DR’s pathogenesis; A literature review was conducted in September of 2021 to explore the therapeutic potential of senolytics in the treatment of DR. Studies that were relevant to the research topic were selected through multiple keyword searches in the search engine, PubMed and thoroughly reviewed using abstracts and full-text articles. We present evidence from animal models for studying cellular senescence in DR and discuss multiple pathogenic mechanisms in cellular senescence and its involvement in DR. We also discuss the current state of pharmaceutical development at preclinical and clinical stages focusing on the senolytic drugs navitoclax, 17-DMAG, piperlongumine, UBX-1325, dasatinib quercetin, and fisetin. In particular, UBX-1325 holds a promising prospect for DR treatment based on the positive outcome of early clinical studies in individuals with diabetic macular edema (DME) and wet age-related macular degeneration.

## 1 Introduction

Diabetic retinopathy (DR) is one of the most common complications of diabetes, affecting 131.66 million people worldwide; this number is projected to grow to 205.32 million by 2045 (Teo et al., 2021). Previous studies have shown that DR is more prevalent in populations from the regions of Africa (35.9%), North America and the Caribbean (33.3%), the Middle East and North Africa (32.9%), Western Pacific (19.2%), Europe (18.75%), Southeast Asia (16.99%), and South and Central America (13.37%) (Sreekumar et al., 2020).

DR is characterized by damage to the vasculature of the eye and, in its later stages, the development of new retinal vasculature. The worsening of DR can lead to intense vision loss and blindness if left untreated. DR consists of four major stages of development: mild non-proliferative DR (NPDR), moderate NPDR, severe NPDR, and the most critical stage, proliferative DR (PDR) (Lechner et al., 2017). The main distinction between the non-proliferative and the proliferative stage is the presence of neovascularization. Neovascularization serves as a mechanism to try and compensate for the damage that has occurred from diabetes to the preexisting retinal vasculature. However, the new blood vessels are abnormal, fragile, perforated, and leaky (Lechner et al., 2017). The development of these abnormal blood vessels ultimately leads to vision loss. Diabetic macular edema (DME) is characterized by a fluid buildup in front of the macula, affecting one in 15 people with diabetes, accounting for 20 million cases worldwide (Tan et al., 2017), and DME is associated with any stage of the DR (Duh et al., 2017).

## 2 Pathogenesis of DR

A combination of genetic, environmental factors and the diabetes milieu itself plays a vital role in the onset of DR and the severity of its progression. Most genes influencing DR onset and progression are components of major pathways such as insulin signaling, neovascularization and angiogenesis, and inflammation (Sharma et al., 2019). Previous studies have also shown that environmental factors such as growing up in a more socially or economically deprived area increase the risk for DR. This could be attributed to a large percentage of unemployed individuals, lack of education, lower rates of reliable transportation, and inadequate access to proper healthcare resources or inability to get to the resources. These environmental factors often lead to a prolonged state of uncontrolled diabetes and can thus heavily increase the risk of developing DR as a complication of diabetes. Along with these socioeconomic and environmental factors that lead to the increased prevalence of DR, there is also a lack of proper monitoring of the visual/retinal state of those with diabetes (Nwanyanwu et al., 2015). This lack of checkups and awareness of DR often progresses much further before being caught. In addition, due to the initial stages of DR being largely asymptomatic, patients do not raise concerns until they are experiencing vision loss which is part of the later stages of DR (Zhang et al., 2013; Nwanyanwu et al., 2015).

**FIGURE 1:**
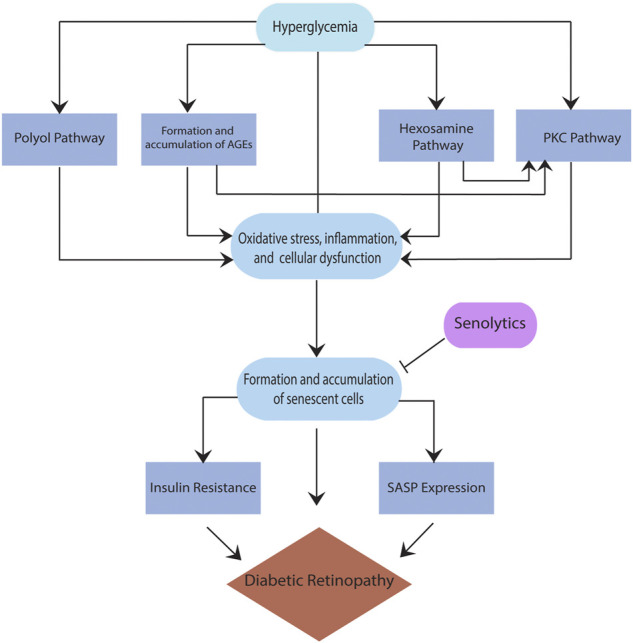
Schematic showing role of cellular senescence in diabetic retinopathy.

While several pathways are found to be critical to the pathogenesis of DR, such as the polyol pathway, the formation and accumulation of advanced glycation end products (AGEs), the plasma kallikrein kinin (PKK) pathway, the hexosamine pathway, and the protein kinase C (PKC) pathway (Safi et al., 2014; Lechner et al., 2017), there is no clear consensus on a particular pathway that has been implicated in DR. Moreover, these pathways are interrelated; for example, sorbitol built up in the polyol pathway accumulates inside the cells, resulting in cellular stress and forming AGEs. When AGEs interact with their receptor for advanced glycation end products (RAGE), they trigger other pathways that lead to the secretion of inflammatory cytokines such as vascular endothelial growth factor (VEGF) (Safi et al., 2014). VEGF overexpression in the retina, specifically in the pericytes, leads to the breakdown of the blood-retina barrier (BRB), which enhances the progression of DR to an advanced form. Also, RAGE activation leads to an increase in reactive oxygen species (ROS), resulting in pericyte and endothelial cell apoptosis, ultimately causing vascular changes and increasing the severity of DR. (Yamagishi, 2011). In addition, the activation of protein kinase C (PKC) either directly or via the hexosamine pathway (Lechner et al., 2017) results in the stimulation of mitogen-activated protein kinase (MAPK), VEGF, plasminogen activator inhibitor-1 (PAI-1), nicotinamide adenine dinucleotide phosphate (NADPH) oxidase, and nuclear factor kappa B (NFkB), all known contributors of inflammation in DR. Regardless of the type of the pathway, hyperglycemia is a critical driver of all above pathways resulting in cellular stress and damage (Figure 1). The reaction to this cellular stress in diabetes and other diseases with increased risk with age is the formation and accumulation of senescent cells, discussed below.

## 3 Cellular senescence

Senescent cells are ‘cells’ that have been irreversibly arrested into a state where they cannot replicate and are resistant to apoptosis; however, they remain in the metabolically active state (Kirkland and Tchkonia, 2017). These cells are irreversibly arrested because they do not respond to external stimuli such as growth factors that could allow them to reenter the cell cycle. The formation of these cells is triggered not only by the cellular stress amassed from the pathways discussed above but also through the metabolic dysfunction induced by hyperglycemia. Typically, the body naturally clears senescent cells through immune system activity, but when these cells accumulate to a certain amount, they cause immune system dysfunction resulting in an inability to remove them (Wissler Gerdes et al., 2020). Once the body reaches a threshold of immune dysfunction, senescence cells accumulate as age and disease state progress. These cells collect specifically in the pathogenic sites of chronic conditions such as neurodegenerative diseases, diabetes, cardiovascular disease, and many others. Many studies have suggested that the formation of senescent cells in response to cellular damage or metabolic dysfunction may initially help prevent apoptosis and neuronal degeneration (Muñoz-Espín and Serrano, 2014; Mosteiro et al., 2016).

While the senescence mechanism may initially be triggered to protect the body from further damage, it inhibits the body from adequately correcting the initial problem causing more damage and progression of the disease. Due to the senescence state, these cells cannot trigger pathways that can help repair the cell, such as reparative angiogenesis. Also, any remodeling that does take place in the cells is faulty due to the accumulation of senescent cells and the inflammatory state they encourage through the secretion of inflammatory factors. This initial protective mechanism can be acute and less detrimental, for example, wound healing response; however, chronic senescence results in a slew of problems (Birch and Gil, 2020). These cells accumulate in the kidneys, retina, pancreas, liver, and adipose tissue in individuals with diabetes (Palmer et al., 2015). The presence and accumulation of senescent cells can increase the risk of diabetes due to insulin resistance.

### 3.1 Mechanisms involving cellular senescence

#### 3.1.1 Tumor suppressor pathways

Senescent cells enter a stable, irreversibly arrested state through the activation of p54/p21^CIP1^ and p16^INK4a^/RB tumor suppressor pathways. Activation of these pathways results from persistent DNA damage response (DDR), causing inhibition of cyclin-dependent kinases required for the cell cycle, thus halting the cell cycle process (Birch and Gil, 2020). DDR is characterized by arresting cell cycle progression until DNA damage is addressed and stopped. This often triggers cells to enter cellular senescence, although it must be noted that cells can enter a senescent state independent of persistent DDR (Herranz and Gil, 2018). A cell must first activate the p53 pathway to enter a senescent state. P53 induces the transcription of the cyclin-dependent kinase inhibitor (CDKi), p21^CIP1,^ which blocks CDK2 activity. The blocking of an essential cyclin-dependent kinase leads to a cellular arrest and the initiation of cellular senescence. This first tumor suppressor pathway triggers short-term cellular senescence/cell arrest and the activation of the second tumor suppressor pathway, the p16^INK4a^/RB pathway, which leads to a sustained irreversible cellular arrest. Persistent cellular stress triggers the p16^INK4a^/RB pathway, activating p16^INK4^, an inhibitor of CDK4 and CDK5. The *CDKN2A* gene that provides instructions for making p16INK4A is a well-studied marker of cell senescence, and its expression levels are doubled during aging (Tabula Muris, 2020). On the contrary, inhibition of important CDKs results in hyperphosphorylation of the retinoblastoma (Rb) gene triggering cell cycle exit and irreversible arrest (Herranz and Gil, 2018).

#### 3.1.2 Paracrine senescence

Paracrine senescence is a process induced by the senescence-associated secretory phenotype (SASP) in which senescent cells can spread senescence to neighboring cells through the expression and release of members of the transforming growth factor-β family (TGF-β), VEGF, and chemokines (Birch and Gil, 2020). This process has been shown in co-culture studies where normal proliferating cells were co-cultured with cells that had undergone induced senescence (Acosta et al., 2013).

#### 3.1.3 Bystander senescence

Bystander senescence is the process of inducing senescence in neighboring cells connected *via* gap junctions. This process’s mechanism is based on interactions between ROS and its associated pathways with other SASP factors such as TGF-β ligands **(** Birch and Gil, 2020). While bystander senescence is well-established in*in vitro* studies, *in vivo* mechanisms of bystander senescence aren’t as well researched and are only inferred to occur based on the clustering of senescent cells in many tissues. However, despite less research in general for *in vivo* bystander senescence, a few studies have been published showing the contribution of bystander senescence *in vivo*. One of these studies conducted in 2018 transplanted human senescent fibroblasts into immunodeficient mice and found that senescence markers levels were significantly higher in myofibres adjacent to the transplanted fibroblasts and much lower in myofibres that were further away (Da Silva et al., 2019), suggesting that bystander mechanism indeed play some role in*in vivo*.

### 3.2 Senescence-associated secretory phenotype

Typically, complications in diabetes arise when the SASP is expressed by senescent cells. SASP consists of an abundance of protein and non-protein signaling molecules, including proteases, ceramides, bradykinins, and damage-associated molecular patterns (DAMPs). When SASP is expressed, a slew of inflammatory factors, growth factors, and cytokines get released, leading to tissue dysfunction and further damage to the tissues (Palmer et al., 2015). SASP is typically activated when macromolecular damage (often DNA) is sensed in the cell. Once this damage is perceived, two primary mediators of the induction of SASP are a retinoic acid-inducible gene I (RIG-I) and inflammasomes. RIG-I is a cytosolic pattern recognition receptor (PRR) responsible for sensing cytoplasmic RNA and leading to senescence-associated inflammation (Acosta et al., 2013). Inflammasomes are another group of pattern recognition specialized to recognize DAMPs and trigger inflammatory cascades or pyroptosis (a form of cell death associated with inflammation) characteristic of SASP expression (Schroder and Tschopp, 2010).

Another critical pathway involved in the induction and regulation of SASP is the cGAS/STING pathway. This pathway is activated when cytosolic DNA is recognized by cyclic GMP-AMP synthase (cGAS). Cytosolic DNA can originate from retrotransposable elements, damaged mitochondrial DNA, and cytosolic chromatin fragments (CFFs). CFFs can arise from senescent cells discarding nuclear chromatin into the cytoplasm of cells, persistent DNA damage response, and dysfunctional mitochondria. Once cGAS senses the cytosolic DNA, it produces GMP-AMP (cGAMP), a secondary messenger that binds to the stimulator of interferon genes (STING) receptor. The STING receptor is an endoplasmic reticulum surface receptor, and through its activation, subsequent activation of tank binding kinase 1 (TBK1), interferon regulatory factor 3 (IRF3), and nuclear factor kappa B (NFkB) occurs. Activation of these factors triggers inflammatory pathways and responses that characterize the expression of SASP (Rodier et al., 2009; Correia-Melo et al., 2016).

## 4 Senescence and diabetes

As established throughout this manuscript, diabetes is a multifactorial disease with many factors combined to induce a diabetic state. Two major risk factors for diabetes, specifically type 2 diabetes (T2D), are age and obesity, which are associated with the increased number of senescent cells. Obesity, specifically abdominal and visceral obesity, can lead to increased inflammation in adipose tissue, triggering the accumulation of senescent cells (Palmer et al., 2019). The increased number of senescent cells helps contribute to the diabetic state due to their generation of insulin resistance in the body. Therefore, the more senescent cells there are, the more insulin resistance the body is experiencing, leading to the development of diabetes. Once the diabetic state is developed, research shows that the elevated glucose and lipid levels in the diabetic microenvironment lead to the formation and further accumulation of more senescent cells (Esser et al., 2014). Due to this, many tissues in diabetes and associated conditions with diabetes are characterized by the accumulation of senescent cells. These tissues and conditions include pancreatic β cells, fatty liver disease, cardiovascular disease, renal dysfunction, cognitive dysfunction, and DR (Palmer et al., 2019). With age, gene expression patterns in the β cells change and increase the expression of genes associated with cellular senescence as cyclin-dependent kinase inhibitors: CDKN2A and CDKN2B (Helman et al., 2016). Mouse models of T1D have shown that clearing senescent β-cells leads to enhanced insulin secretion, suggesting a link between cellular senescence in β-cells and insulin deficiency (Thompson et al., 2019).

Increased risk for non-alcoholic fatty liver disease (NAFLD), a condition characterized by severe fat buildup in the liver, is also associated with T2D. Previous studies suggest that with the increasing severity of NAFLD, an increase in senescent cells in the liver and senolytic treatment helps reduce the severity of NAFLD (Ogrodnik et al., 2017). Cardiovascular disease, renal dysfunction, and cognitive dysfunction are all associated with increased cellular senescence burden with age, obesity, and in individuals who have diabetes. Accumulating evidence suggests that clearance of senescent cells in all of these conditions can greatly improve the health of individuals affected by them (Palmer et al., 2019).

## 5 Senescence and DR

As previously discussed, senescence plays a major role in many diabetes-related complications, including DR. Sustained hyperglycemic conditions in the body lead to the formation and accumulation of senescent cells, which further contributes to this accumulation of senescent cells (Esser et al., 2014). The senescence of retinal endothelial cells mainly characterizes senescence in DR. These senescent cells express SASP and contribute to angiogenesis in DR (Donato et al., 2015). SASP triggers pathological angiogenesis through the expression of growth factors and inflammatory cytokines. These factors lead to the neovascularization spreading of senescence through paracrine senescence (Oubaha et al., 2016). Senescent cells in DR also prove to be an obstacle for reparative mechanisms leading to the leaky and fragile vessels formed during neovascularization.

### 5.1 Preclinical studies

Several preclinical studies in the last 5 years have established a significant link between senescence and DR (Shosha et al., 2018; Binet et al., 2020; Gericke et al., 2021). One such study examined this link by creating a mouse model of oxygen-induced retinopathy (OIR) that exhibits ischemic avascular zones and neovascularization similar to what would be seen in PDR (Binet et al., 2020). This study’s main findings were the presence of neutrophils during neovascularization and their migration toward neovascular tufts. This finding is relevant because PDR individuals often exhibit migration of neutrophils, perhaps due to the production of neutrophil extracellular traps (NETs). NETs promote apoptosis of senescent cells in the vasculature of the retina and set up the retina to undergo reparative vascularization, ultimately benefiting the retina’s overall health (Binet et al., 2020).

Similar to the above *in vivo* findings, another preclinical study assessed the pharmacological effect of sulodexide, a highly purified mixture of glycosaminoglycans, on glucose-induced senescent cell burden in human retinal endothelial cells (HRECs) (Gericke et al., 2021). HRECs undergoing replicative senescence in the presence of high glucose or mannitol (osmotic control) when treated with sulodexide exhibited a decrease in inflammatory markers with an ultimate reduction in the senescent cell burden (Gericke et al., 2021).

### 5.2 Clinical studies

Along with preclinical evidence of the relationship between cellular senescence and DR, there have also been strides in clinical studies to provide further evidence for their relationship and highlight cellular senescence as a potential therapeutic target for treating DR (Oubaha et al., 2016; Crespo-Garcia et al., 2021). One study assessing the clinical relevance of this relationship was conducted by retrieving samples of undiluted vitreous fluid from patients afflicted with active PDR and retrieving samples from patients with nonvascular retinal damage unrelated to diabetes. The vitreous fluid of the participants was analyzed for SASP proteins through multiplex magnetic bead-based immunoassays. The results of this analysis showed an extremely significant increase (*p* < 0.01 and *p* < 0.001) in SASP-associated cytokines PAI-1, interleukin-6 (IL-6), IL-8, and VEGF in the patients with active PDR compared to the control group (Oubaha et al., 2016).

Another study examining the retinas of post-mortem patients with PDR found more than 2.2 times the amount of cells expressing p16^INK4A^ (a measure of senescence due to it being an indication of cell-cycle arrest) compared to the retinas of the non-diabetic controls (Crespo-Garcia et al., 2021). Both of the previous study’s results indicate that senescent cell burden has a significant role in DR, further suggesting that clearance of senescent cells can be an extremely beneficial therapeutic target for preventing the progression of DR and improving vision and overall health in patients afflicted with this condition.

## 6 Senolytics

Senolytics are a class of drugs that clear senescent cells (cells resistant to apoptosis and non-dividing but are still metabolically active) from various tissues in the body without harming or disrupting healthy tissues (Wissler Gerdes et al., 2020). This potential therapeutic option could revolutionize the treatment of many age-related and chronic diseases to slow disease progression and improve patients’ overall health. Clearing senescent cells will also decrease the expression of SASP and other senescence-associated mechanisms in the body, leading to a lower degree of disease-related complications (Ge et al., 2021). Senolytic drugs are unique compared to current treatments for chronic and age-related diseases. They go beyond symptom management and do not have many adverse effects. In addition, senolytics are administered intermittently because senescent cells take multiple weeks to develop (Kirkland and Tchkonia, 2020). Although senolytics do not stop the production of senescent cells, their intermittent administration helps clear them over time. A summary of senolytic candidate molecules is outlined in Table 1.

**TABLE 1:** Summary of senolytic candidate molecules.

Drug	Type	Cell Target	Advancement	Risks/side effects
Navitoclax	Bcl-2 inhibitor	HUVECs Hematopoietic Stem cells	Preclinical candidate	Bleeding, thrombocytopenia, and neutropenia(Chang et al., 2016; Prasnikar et al., 2021)
17-DMAG	Heat shock protein 90 inhibitor	Progeroid mouse model and Mouse embryonic fibroblast cells	Preclinical candidate	N/A (Fuhrmann-Stroissnigg et al., 2017)
Piperlongumine (PL)	Binds to OXR 1, recruits’ proteasome to force apoptosis	WI-38 Fibroblast Cells	Preclinical candidate	N/A
Dasatinib (D) and Quercetin (Q)	D = tyrosine kinase inhibitor O = flavonoid	Adipose tissue	Clinical candidate	No serious adverse reactions requiring hospitalization, kidney injury requiring dialysis, or death and discontinuation of drug (Hickson et al., 2019)
Fisetin	Flavonoid	Articular cartilage	Clinical candidate	Ongoing (no side effects in previous studies) (Yousefzadeh et al., 2018)
UBX-1325	BCL-xL inhibitor	Vasculature	Clinical candidate	well-tolerated, two events of a decrease in best corrected visual acuity (BCVA) (Seeking Alpha, 2022)

### 6.1 Preclinical candidates

#### 6.1.1 Navitoclax (ABT-263)

Navitoclax is a small molecule B-cell lymphoma 2 (Bcl-2) inhibitor. Navitoclax selectively binds to pro-apoptotic proteins, specifically BCL-xL, BCL-2, and BCL-w, preventing them from binding to Bak and Bax’s apoptotic effectors. These properties of navitoclax reduce the number of cells that avoid apoptosis in a cancerous or senescent state (Zhu et al., 2020). In rodent studies, navitoclax helped delay the progression of atherosclerosis and neurodegeneration due to the effective clearing of senescent cells (Sreekumar et al., 2020). While navitoclax can be an effective senolytic candidate, *in vitro* studies have shown an increase in cytotoxicity primarily through Bcl-2-induced neutrophil toxicity. In addition, navitoclax exhibits a cell-specific effect; for example, it clears senescent cells from human umbilical vein endothelial cells (HUVECs), however does not work on human preadipocytes (Zhu et al., 2016). Navitoclax has been shown to cause bleeding by leading to thrombocytopenia, neutropenia, and bleeding (Prasnikar et al., 2021). Navitoclax has not progressed to clinical trials yet, possibly due to the more severe adverse effects of the drug.

#### 6.1.2 Heat shock protein 90 inhibitors

HSP90 inhibitors work by downregulating the functions of HSP90 in an anti-apoptotic pathway. This is extremely useful in the case of senescent cells, where anti-apoptotic pathways are heavily upregulated to avoid cell death. One specific HSP90 inhibitor named 17-DMAG is an analog of geldanamycin, a benzoquinone antineoplastic antibiotic that binds to HSP90 to prevent its functions. Geldanamycin has been shown to improve the overall health of a mouse model of human progeroid syndrome by reducing and delaying the onset of age-related conditions (Sreekumar et al., 2020).

#### 6.1.3 Piperlongumine

This drug is a biologically active alkaloid and a nitrogen-containing compound derived from the *Piper longum Linn* plant. PL has been shown in several studies to have promising anticancer benefits and was identified as a senolytic (Liu et al., 2018). PL selectively targets senescent cells and binding oxidation resistance 1 (OXR 1). By binding to OXR1, piperlongumine recruits ubiquitin-proteasome to force the senescent cells to undergo apoptosis (Wang et al., 2016).

#### 6.1.4 UBX-1967

UBX-1967 is a potent senolytic member of Bcl-2 family. This agent has been tested in the OIR model, where a single intravitreal administration of UBX-1967 decreased cellular senescence and SASP related genes such as *Cdkn2a(Ink4a)*, *Cdkn1a*, *Serpine1,* and *TNF*, interestingly there was no effect on VEGF. Furthermore, UBX-1967 also enhanced vascular regeneration (Crespo-Garcia et al., 2021).

### 6.2 Clinical candidates

#### 6.2.1 Dasatinib and quercetin

D + Q drug combination was the first senolytic treatment discovered. Dasatinib (D) is a tyrosine kinase inhibitor and an antineoplastic agent commonly used to treat certain cancers such as leukemia. Dasatinib’s mechanism of action is targeted, which clears damaged cells without harming healthy cells. Quercetin (Q) is a flavanoid, an antidoxidant present in many fruits and vegetables. The combination of D + Q as a senolytic treatment effectively reduced the senescent cell burden in the mouse model for the human progeroid syndrome (Zhu et al., 2015).

Since D + Q was the first senolytic treatment discovered, it was also the first to progress to clinical trials. An open-label phase 1 pilot study was conducted to test the efficacy of D + Q treatment on individuals with diabetic kidney disease. The subjects were administered 100 mg of D and 1,000 mg of Q for 3 days. Samples of adipose tissue, skin biopsies, and blood were collected before drug administration and 11 days after administration to compare the treatment’s senolytic effect. This study showed that D + Q treatment decreased the number of senescent cells in adipose tissue and decreased cells expressing tumor suppressor protein, p16^INK4A^, and cyclin-dependent kinase inhibitor 1, p21^CIP1^. In addition, SASP factors were reduced after treatment with D + Q. These results support the therapeutic potential of D + Q (Hickson et al., 2019).

#### 6.2.2 Fisetin

Fisetin is another flavonoid and antioxidant like quercetin. In animal studies treating progeroid and old mice with the senolytic drug, fisetin reduced senescence biomarkers throughout the body. In addition, fisetin helped restore homeostasis in dysregulated tissues, reduced age-related conditions, and consequently extended the lifespan of the mice (Yousefzadeh et al., 2018).

Fisetin is undergoing a phase 2 double-blind placebo-controlled trial in osteoarthritis-related articular cartilage degeneration. Fisetin will be given orally as 100 mg capsules for 2 days, followed by a treatment-free period of 28 days. The second round of fisetin will be administered after 28 days off (Prasnikar et al., 2021). The study is estimated to be completed by December 2022 (https://clinicaltrials.gov/ct2/show/NCT04210986?cond=fisetin&draw=2&rank=8).

#### 6.2.3 UBX-1325

UBX-1325 is an inhibitor of a pro-apoptotic protein, BCL-xL. UBX-1325 is a unique senolytic because it is tailored to combat senescence associated with age-related diseases of the eye such as PDR, age-related macular degeneration (AMD), age-related macular edema, and other diseases involving vascular dysfunction of the eye. UBX-1325 has been shown to selectively eliminate senescent cells *in vitro* and inhibit retinal neovascularization, reducing vascular leakage in animal models (Unitybiotech, 2018). In addition, UBX-1325 has been shown to promote apoptosis of senescent cells in OIR mouse model. UBX-1325 also enhances the actions of anti-VEGF agents in the OIR model, thus helping down-regulate SASP and senescence in general (Unitybiotech, 2018). UBX-1325 also improves retinal vasculature in an animal model of OIR and streptozotocin (STZ) induced diabetes (Tsuruda et al., 2021). Phase 1 studies for UBX-1325 have been recently completed for patients with DME and AMD (Hutton, 2021). Using a single dose of UBX-1325, there was an overall improvement in visual acuity through 24 weeks. Overall, UBX-1325 was well tolerated without signs of intraocular inflammation and other ocular adversities. The phase 2 clinical studies of UBX-1325 are underway (DME, 2022).

## 7 Conclusion

DR is a multifactorial disease for which there is no current treatment. Considering the blinding nature of DR’s progression development of newer treatments is critical. Due to the long-standing and progressive nature of DR, senescent cells continually accumulate during DR; in particular, SASP triggers a release of a variety of cytokines such as interleukins and VEGF. In addition, pathological vasculature of the retina in DR engages cell cycle inhibitory protein p16 ^INK4A^ and anti-apoptotic protein, BCL-xL, which together limit the retina’s ability to clear senescent cells of the retina. Efficient clearing of p16^INK4A^ cells suppresses pathological angiogenesis. Therefore, pharmacological treatment with senolytics is promising for treatment in DR. Table 1 summarizes the current preclinical and clinical candidates of senolytics in the treatment of DR. Although there are currently little to no reported risks for the majority of senolytics discussed, it is critical to note that many of these drugs are still in their initial experimental stages. The actual risks of senolytics could not be fully assessed until more of these candidates move on or progress further into their clinical studies. However, the lack of reported risks at the moment may be an encouraging sign for using senolytics.

While various pharmacological targets such as D + Q, and fisetin have reached the clinical phase, there are no ongoing trials in ocular diseases. UBX-1967 has been shown to be effective in pathological neovascularization of OIR. UBX-1325 is a forerunner for ocular treatment and has improved visual outcomes for individuals with AMD and DME. Phase 2a studies for UBX-1325 in DME are underway and expected to be completed by October 2022. A positive outcome of these studies holds excellent potential for treating DR outcomes with the added advantage of fewer injections.
